# Improving diet, self-rated health, & BMI for older adults with a student-led health education intervention

**DOI:** 10.1093/geroni/igaf122.126

**Published:** 2025-12-31

**Authors:** Britteny Howell, Amber Worthington, Leslie Redmond, Vanessa Hiratsuka

**Affiliations:** University of Alaska Anchorage, Anchorage, Alaska, United States; University of Alaska Anchorage, Anchorage, Alaska, United States; University of Manitoba, Winnipeg, Manitoba, Canada; Southcentral Foundation, Anchorage, Alaska, United States

## Abstract

Older adults in Alaska make up the fastest growing population in the state and face some of the greatest provider shortages in the country. Research suggests that older adults may respond better to positive, hopeful information that aligns with their desires and goals for the future. Therefore, we designed and delivered a 15-week, student-led health intervention using Persuasive Hope Theory to 39 older adults to increase feelings of hope and improve health. Our research question was: Did self-efficacy, fruit and vegetable intake, physical activity, self-rated health, or BMI improve significantly as a result of the program? Dependent sample t-tests revealed that the number of servings of fruit and vegetables consumed increased significantly after the program compared to before the program (p<.01). BMI was also statistically significantly lower after the program compared to before the program (p<.05) and participants’ self-rated health was significantly higher after the program compared to before the program (p<.01). There was no significant difference in self-efficacy or physical activity levels over time. A power analysis was conducted using G*Power version 3.1.9.7 that indicated the required sample size to achieve 80% power at a significance criterion of α = 0.5, is N = 27 for a dependent sample t-test. Despite the use of a small, convenience sample, results indicate that participants were satisfied with the student-led intervention that significantly helped to decrease BMI and increase fruit and vegetable intake and self-rated health among the sample population, which can serve as a model for other hope-based, intergenerational healthy aging initiatives.

